# An *in vitro* Perfused Macroencapsulation Device to Study Hemocompatibility and Survival of Islet-Like Cell Clusters

**DOI:** 10.3389/fbioe.2021.674125

**Published:** 2021-05-28

**Authors:** Stephanie A. Fernandez, Lisa Danielczak, Gabrielle Gauvin-Rossignol, Craig Hasilo, André Bégin-Drolet, Jean Ruel, Steven Paraskevas, Richard L. Leask, Corinne A. Hoesli

**Affiliations:** ^1^Department of Chemical Engineering, McGill University, Montréal, QC, Canada; ^2^Department of Mechanical Engineering, Laval University, Québec City, QC, Canada; ^3^Department of Surgery, McGill University Health Centre, Montréal, QC, Canada; ^4^Department of Biomedical Engineering, McGill University, Montréal, QC, Canada

**Keywords:** type 1 diabetes, alginate-based encapsulation, pancreatic islet hypoxia, perfused vascular prosthesis, convective mass transport

## Abstract

Transplantation of hydrogel-encapsulated pancreatic islets is a promising long-term treatment for type 1 diabetes that restores blood glucose regulation while providing graft immunoprotection. Most human-scale islet encapsulation devices that rely solely on diffusion fail to provide sufficient surface area to meet islet oxygen demands. Perfused macroencapsulation devices use blood flow to mitigate oxygen limitations but increase the complexity of blood-device interactions. Here we describe a human-scale *in vitro* perfusion system to study hemocompatibility and performance of islet-like cell clusters (ILCs) in alginate hydrogel. A cylindrical perfusion device was designed for multi-day culture without leakage, contamination, or flow occlusion. Rat blood perfusion was assessed for prothrombin time and international normalized ratio and demonstrated no significant change in clotting time. *Ex vivo* perfusion performed with rats showed patency of the device for over 100 min using Doppler ultrasound imaging. PET-CT imaging of the device successfully visualized metabolically active mouse insulinoma 6 ILCs. ILCs cultured for 7 days under static conditions exhibited abnormal morphology and increased activated caspase-3 staining when compared with the perfused device. These findings reinforce the need for convective transport in macroencapsulation strategies and offer a robust and versatile *in vitro* system to better inform preclinical design.

## Introduction

Type 1 diabetes is an autoimmune disease that selectively destroys the glucose-sensing, insulin-secreting islet beta cells. Patients are typically treated via frequent blood glucose monitoring and administration of exogenous insulin. By contrast, islet transplantation aims to provide long-term blood glucose sensing and regulation by replacing the missing beta cells. Current islet transplantation procedures require lifelong immunosuppression to minimize graft rejection, although chronic rejection is not completely avoided (Korsgren et al., 2005; Gibly et al., 2011; McCall and Shapiro, 2012). Lifelong immunosuppression is also associated with increased risks of infection or cancer (Ryan et al., 2004). As an alternative, numerous micro- or macroencapsulation methods have been proposed to safely deliver the therapeutic cells while protecting them against host immune attack (Scharp and Marchetti, 2014; Moeun et al., 2019). Microencapsulation devices each contain one or a few islets (Scharp and Marchetti, 2014), while macroencapsulation approaches aim to deliver an entire therapeutic cell dose in one or a few devices.

Macroencapsulation strategies are attractive in that they allow for better control over device localization and possible retrieval if needed. However, the scale of such devices impedes adequate mass transport of oxygen and nutrients via passive diffusion (Scharp and Marchetti, 2014). Hypoxic zones within the device often lead to impaired cell function, cellular necrosis, and graft failure (de Vos et al., 1999; Dulong and Legallais, 2007). There is an increasing body of work centered on device prevascularization and hybrid micro/macroencapsulation strategies to improve graft survival. For example, Mridha et al. (2020) have developed a prevascularized device for subcutaneous implantation that consists of polycaprolactone scaffolds housing alginate-microencapsulated islets. While this and other designs have shown promising blood glucose normalization results in rodents, effective scale-up for therapeutic use in humans has yet to be demonstrated (Song and Roy, 2016; Desai and Shea, 2017; Moeun et al., 2019).

Another way to address oxygen limitations in islet macroencapsulation systems is to design perfused vascular prostheses. Petruzzo et al. (1991) proposed a vascular prosthesis loaded with islets microencapsulated in alginate-polylysine. Song et al. (2017) take another approach, proposing a silicon nanopore membrane (SNM) device consisting of an islet-agarose gel featuring ultrafiltrate channels. The performance of these vascular devices is typically tested in larger animals such as dogs and pigs to better reflect oxygen limitations and blood flows expected in humans. However, the cost and complexity of these animal studies limit throughput. It is vital to develop robust *in vitro* platforms to study device-blood interactions and cell survival in human-scale vascularized macroencapsulation systems to accelerate device optimization and translational efforts. Currently, 3D *in vitro* cell culture systems focus on establishing suitable scaffold materials that mimic the extracellular matrix and promote spheroid growth, cell differentiation, and various cell interaction models (Ravi et al., 2015). However, there remains a gap in mass transport strategies to adequately nourish thick tissue constructs.

Here we present the dynamics of an *in vitro* macroencapsulation device that employs convective mass transport to meet the high oxygen demand of hydrogel-immobilized pancreatic beta cells. Alginate has been the forerunner among immunoprotective materials for islet microencapsulation (de Vos et al., 2014), as well as explored for various macroencapsulation strategies, such as single or multi-layered sheets (Storrs et al., 2001; Scharp and Marchetti, 2014). The *in vitro* perfusion culture system allows for an in-depth investigation into oxygen effects within a three-dimensional environment, as well as an assessment of the glucose-insulin kinetics to contribute to the discussion surrounding beta cell engraftment and function. This system can also be used to investigate primary islets from animal models including pigs and humans, providing a more complete look at islet cell performance to better inform preclinical design. This manuscript describes the perfusion system and proof of concept of its application to study hemocompatibility and survival of islet-like cell clusters (ILCs). The long-term goal of this platform is to accommodate human-scale cell doses up to 1 million islet equivalents (IEQ) to evaluate therapeutic efficacy *in vitro*.

## Materials and Methods

### *In vitro* Device

**FIGURE 1:**
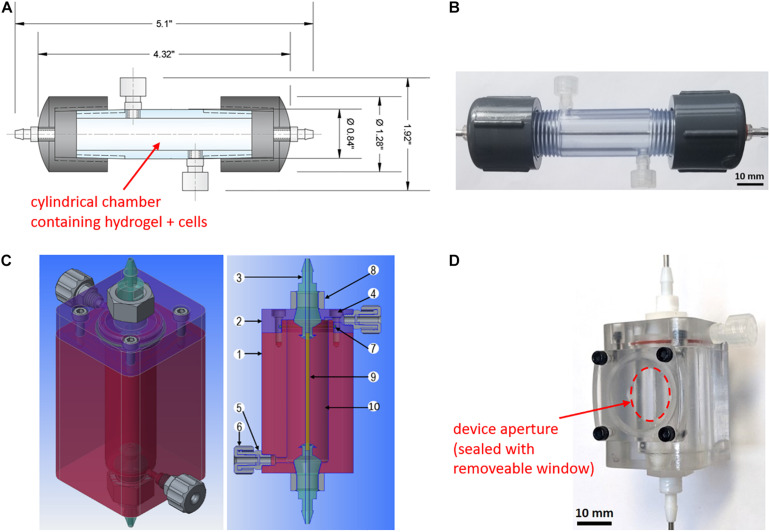
*In vitro* and *ex vivo* perfusion device designs. **(A)** *Device 1* two-dimensional computer-aided design generated using Autodesk AutoCAD software. The device capacity is 18 mL. **(B)** Assembled *Device 1* without gel cast and containing a 2-mm stainless steel rod as a vascular template. **(C)** *Device 2* three-dimensional computer-aided design generated using PTC Creo software. The device capacity is 7.2 mL. Components are as follows: (1) hollow polycarbonate chamber; (2) polycarbonate end cap; (3) custom polytetrafluoroethylene barbed connector; (4) nylon socket head cap screws; (5) filling and overflow port; (6) polypropylene Luer fitting and cap; (7) silicone O-ring; (8) custom nylon hex nut; (9) vascular template; (10) hydrogel with immobilized cells. **(D)** Assembled *Device 2* without gel cast and containing a 1.6-mm stainless steel rod as a vascular template. A removable lid secured with four screws allows for the placement of an ultrasound transducer through a device aperture (indicated in red) to monitor fluid flow through the channel.

Device 1 (Figures 1A,B) was designed for human-scale *in vitro* experimentation and assembled using readily available parts from McMaster-Carr and Cole-Parmer in standard sizes. With a single-channel vascular template, the device capacity is 18 mL, which can accommodate a typical therapeutic dose of islets for one adult patient (Hering et al., 2016) at a density of 30,000–40,000 IEQ/mL hydrogel. The vascular template is a negative mold present during hydrogel casting and subsequently removed to leave hollow, perfusable channels mimicking vasculature. The body of the device is composed of a polyvinyl chloride (PVC) threaded pipe nipple and two PVC caps. Threaded holes were added to accommodate fittings for fluid flow and gel casting. Polycarbonate barbed tube-to-threaded fittings used with silicone O-rings were placed at either PVC cap to create a perfusion inlet and outlet. Along the device body, two polypropylene Luer fittings and caps serve as gel filling and overflow ports. A 2-mm diameter stainless steel rod was fit through the inlet and outlet connectors to act as a vascular template (Figure 1B), leaving space for a 5.4 mm-thick gel between the perfusion channel and the device wall.

Device 1 was modified to perform *ex vivo* perfusion studies using a rat model. The capacity was downsized by a factor of 2.5 to 7.2 mL containing a perfusion channel of 1.6 mm in diameter (Device 2, Figure 1C). The device housing was redesigned to have a flat base and an aperture to provide access to the hydrogel and accommodate an ultrasound transducer (Figure 1D). Device 2 was fabricated using a computer numeric controlled (CNC) mill and lathe. The device body is composed of a hollow polycarbonate chamber and end cap, with custom polytetrafluoroethylene (PTFE) fittings creating the device inlet and outlet and two side ports added for gel filling as further detailed in Figure 1C.

### Flow Loop and Sterilization

An *in vitro* flow loop was designed around a peristaltic pump. A 100-mL glass bottle was used as a perfusate reservoir with silicone liquid inlet and outlet lines as well as a third opening for sterile gas exchange using a 0.45 μm PTFE filter (#RK-29550-10, Cole-Parmer). The flow loop was created using silicone tubing (#RK-07625-48, Cole-Parmer) except for the segment running through the peristaltic pump, which consisted of PharMed^®^ BPT tubing (#RK-95723-48, Cole-Parmer) connected to the silicone loop via polypropylene tube-to-tube fittings.

The medium reservoir, tubing loop, metal rod, and Device 2 components (aside from the nylon screws) were pre-assembled (uncapped to allow steam entry and exit) and sterilized by autoclaving in a gravity cycle at 121°C for 30 min. Threaded fittings were wrapped with Teflon tape to create a tight seal. The PVC device body (Device 1) was sterilized by soaking in 100% ethanol for 60 min and left to dry overnight in a biological safety cabinet prior to completing device assembly.

### Gel Casting and *in vitro* Perfusion

A 2.5% alginate stock solution (Manugel GHB, FMC Biopolymer) was prepared in pH 7.4 HEPES-buffered saline (Fisher BioReagents #BP310 and #BP3581, Thermo Fisher Scientific). A 0.5 M calcium carbonate stock suspension (CaCO_3_; Avantor #1301-01, VWR) was prepared in pH 7.4 HEPES-buffered saline. Both stocks were sterilized by autoclaving at 121°C for 30 min. Glucono-δ-lactone (GDL; Sigma-Aldrich #G4750, MilliporeSigma) was dissolved in HEPES-buffered saline and sterilized through a 0.2 μm nylon syringe filter (Fisherbrand^TM^ #09-719C, Thermo Fisher Scientific) immediately prior to use. The alginate hydrogel mixture was prepared for casting using the above reagents and culture medium (with or without cells) to produce final gel concentrations of 2% alginate, 30 mM CaCO_3_, and 60 mM GDL. A 9:1 volume ratio of alginate-to-medium was maintained. The medium used was Dulbecco’s Modified Eagle medium (DMEM; Gibco #10313-021, Thermo Fisher Scientific), while cell suspensions were prepared as described below.

To initiate internal gelation, GDL was mixed in immediately prior to casting. The gel mixture was loaded into a syringe and cast in a perfusion device via its Luer filling and overflow ports. The device was then incubated at room temperature for 60 min to ensure adequate gelation. The 2-mm metal rod vascular template was subsequently removed and the device was connected to a tubing loop for perfusion culture. *In vitro* device perfusion was established using a peristaltic pump set to a flow rate of 3 mL/min to mimic laminar blood flow through a medium to small artery 2 mm in diameter (Reynolds number, Re <50). The medium was drawn from a reservoir containing 30 mL of DMEM. The entire setup (reservoir, flow loop, device) was placed inside an incubator at 37°C and 5% CO_2_ except for studies with rat blood.

### *In vitro* and *ex vivo* Rat Blood Perfusion

All animal studies were approved by the McGill University Animal Care Committee (UACC; protocol #7461 and #2016-7865). Rat blood was circulated through devices using either an external pump or cannulated rats. When using an external pump, rat blood was collected in sodium citrate tubes (BD #369714) from 12 male Lewis rats > 9 months old. Device 1 was cast with cell-free 2% alginate and perfusion loops were rinsed with a 4% sodium citrate solution prior to loading 30 mL of blood. Four conditions were tested at room temperature: (i) perfusion, with device; (ii) perfusion, tubing only; (iii) static, with device; and (iv) static, tubing only. These conditions were replicated with three independent blood collections. Plasma was isolated from whole blood samples collected immediately before (*t* = 0) and after 2-h perfusion using centrifugation (1,500 rpm for 15 min), then frozen and sent to IDEXX Reference Laboratories to measure prothrombin time and international normalized ratio (INR) using a Stago STA Satellite automated coagulation analyzer.

For perfusion using animals as a direct blood source, 12 male Lewis rats weighing 400–606 g (weight measured for 8 out of 12 animals) were heparinized and cannulated at either the femoral artery or the carotid artery. Four animals were excluded from analysis due to premature death during surgery or failed cannulation of the artery. Devices (Device 2) were cast with cell-free 2% alginate. Perfusion loops were rinsed with 200 IU of heparin per mL of 0.9% saline (#C504805, Fresenius Kabi) prior to connecting them to the catheter. Perfusion was run up to 90 min or until the failure of the animal. To supplement the *in vitro* rat blood perfusions previously performed, one animal connected to tubing only was monitored for 135 min to observe any severe adverse effects, such as acute thrombosis or animal failure, in the absence of a perfusion device. As this was not the case, tubing-only conditions were omitted from the next animal studies. Three animals were monitored over an abridged perfusion period (40–85 min) due to surgical difficulty achieving a successful cannulation. Four animals were monitored over a perfusion period above 90 min (range of 105–218 min). Upon establishing blood flow throughout the loop, the device aperture was opened and Doppler ultrasound readings (VisualSonics Vevo 2100 with Micro Scan transducer MS250, 13–24 MHz) were acquired to assess flow rate.

### Adherent Cell Culture

Mouse insulinoma 6 (MIN6) pancreatic beta cells were graciously received from Dr. James D. Johnson, University of British Columbia (originally obtained from Dr. Jun-ichi Miyazaki, Osaka University). MIN6 cells were seeded at 4–8 × 10^5^ cells/cm^2^ in tissue culture treated polystyrene cell culture flasks in complete medium consisting of DMEM supplemented with 10% fetal bovine serum (FBS; HyClone^TM^ #SH3039602, Thermo Fisher Scientific), 1% penicillin/streptomycin (Gibco #15140-122, Thermo Fisher Scientific), 1% L-glutamine (Gibco #25030-081, Thermo Fisher Scientific), and 0.1% 2-mercaptoethanol (Fisher Chemical #O3446I, Thermo Fisher Scientific). Cells were cultured at 37°C and 5% CO_2_ with medium changes every 48 h. At 90% confluency, cells were passaged using 1X TrypLE Express Enzyme (Gibco #12605-028, Thermo Fisher Scientific).

### Encapsulation and Culture of Islet-Like Clusters

MIN6 cells were passaged a minimum of twice before seeding at 1.2 × 10^6^ cells/macrowell in AggreWell^TM^400 24-well plates (#34415, STEMCELL Technologies) and cultured for 48 h in complete medium at 37°C and 5% CO_2_ to generate islet-like clusters (ILCs) in the 100–200 μm diameter range. This size was selected to generate approximately 1,000 beta cells per ILC, corresponding to the beta cell content in native human islets (Pisania et al., 2010). Seeding and harvesting steps were performed in accordance with the AggreWell^TM^400 manufacturer’s protocol and live/dead staining was performed on sample ILCs to qualitatively verify cell survival (data not shown). Harvested ILCs were immediately encapsulated in alginate and cast in Device 1 to achieve a final concentration of 1,000 ILCs/mL, followed by perfused culture inside an incubator. For multi-day cultures, the medium in the reservoir was refreshed every 72 h.

In the case of static culture conditions, the cast device was connected to short pieces of tubing at the inlet and outlet. The perfusion channel was manually loaded with culture medium using a syringe and the tubing ends were sealed with 0.45 μm sterile PTFE filters. The static devices were incubated at 37°C and 5% CO_2_. Culture medium was refreshed every 24 h by manually removing old medium and loading fresh medium into the perfusion channel using a syringe.

### PET-CT Imaging

A PET-CT imaging method was developed to visualize beta cell metabolic activity within Device 1. Four static and four perfused devices were prepared from independent cell stocks (*N* = 4 per condition). MIN6 ILCs of 100–200 μm diameter were generated using AggreWell^TM^400 plates and encapsulated at a density of 1,000 ILCs/mL alginate gel. After culturing for 3–7 days at 37°C and 5% CO_2_, culture medium was evacuated from the perfusion loop and fludeoxyglucose (FDG), a radiolabeled glucose analog, was manually loaded by syringe into the perfusion channel (2 MBq injection). After incubating under static, room temperature conditions for 90 min, the device was connected to tubing and flushed with fresh culture medium for 28 min to remove any unbound FDG, followed by flushing with air for 2 min to remove traces of liquid in the perfusion channel. The device was imaged using a Mediso nanoScan PET/CT (scan time 90 min). The gel was then removed from the device and fixed for 24 h at room temperature in a modified Bouin’s solution composed of 3.2 M formaldehyde (Fisher BioReagents #BP531, Thermo Fisher Scientific) and 0.83 M glacial acetic acid (Fisher Chemical # FLA38500, Thermo Fisher Scientific) in reverse osmosis water. VivoQuant^TM^ post-processing software was used to generate a three-dimensional representation of metabolic activity within the scanned device. AMIDE 1.0.4 Medical Image Data Examiner software was used to quantify the mean FDG activity in each device. This was done by taking sections orthogonal to the perfusion channel and calculating the statistics of the region of interest (ROI). Per device, 9 sections (13 mm diameter, 0.4 mm thickness) were taken at 5-mm intervals near the middle of the device.

### Histology and Staining

At the end of the culture period, gel samples were removed from the devices (Device 1) and fixed in modified Bouin’s solution for 24 h at room temperature. Segments 3 cm in length were taken from the middle of each sample (corresponding to the middle of the device) and sent for paraffin embedding, sectioning, deparaffinization and staining (core facility of the Research Institute of the McGill University Health Center). Sections of 6 μm thickness were taken orthogonal to the channel direction followed by automated hematoxylin and eosin (H&E) and cleaved caspase-3 staining. Per device, 3 H&E sections and 3 cleaved caspase-3 sections were generated and analyzed using light microscopy (*N* = 3 biological replicates per condition). Images were analyzed for ILC size and staining intensity using ImageJ software.

### Statistical Analysis

INR data was analyzed via the non-parametric Kruskal-Wallis test comparing the medians of all sample conditions. This was followed by the Wilcoxon-Mann-Whitney test to compare the median of the original (*t* = 0) blood sample with each of the other sample conditions. All statistical analysis was conducted using GraphPad Prism 5.01 software with α = 0.05.

FDG activity was calculated using AMIDE 1.0.4 Medical Image Data Examiner and exported to GraphPad Prism for statistical analysis and graphing of the mean and standard deviation. Relative differences were calculated in Microsoft Excel. ILC size and histological staining data were collected using ImageJ and further analyzed in Microsoft Excel and GraphPad Prism for the mean and standard deviation. Staining intensities were compared through a one-way analysis of variance (ANOVA) with a two-tailed *t*-test assuming equal variances (verified through an *F*-test).

## Results

### Device Design

Table 1 summarizes the main design considerations for developing the proposed *in vitro* macroencapsulation device. Devices 1 and 2 were designed as cylinders to minimize acute angles and facilitate future analysis of a radial mass transport profile between the perfusion channel and the outer edge of the gel (Figure 1). Use of standard parts and fittings was maximized for Device 1, where a capacity of 18 mL was selected. However, the device body may also be custom fabricated to modify the capacity as needed. Device 2 (Figures 1C,D), designed for ex vivo studies, was constructed with a flat base and side aperture to facilitate Doppler ultrasound imaging. The capacity was scaled down along with channel and tubing size to reduce the total volume of blood to <1.5 mL circulating in the *ex vivo* flow loop. The devices were designed at human scale to accommodate cell seeding densities up to several thousand islets or ILCs per mL of gel, permitting investigation of cell performance at both low and therapeutic cell doses. In this work, a low cell seeding density of 1,000 ILCs/mL gel was chosen as a starting point while testing out various methodologies for analyzing device performance.

**TABLE 1:** Design considerations for a perfused *in vitro* macroencapsulation device.

Category	Design criterion	Comments
Materials	Easily sterilized (preferably autoclavable)	Compatible with a typical autoclave cycle (121°C) without affecting machined features such as threading with repeated exposure to autoclave cycles. Does not react with steam additives or residues in the autoclave.
	Cytocompatible	Suitable for incubation at 37°C in a humid environment for several weeks at a time.
	Optically clear	Ideal to visually monitor device loading and perfusion.
General flow and pressure considerations	No acute angles, contraction or expansion in the flow path (connectors, adaptors, tubing)	Avoid features conducive to high shear stresses and flow instabilities, recirculation, or stagnation of flow.
	Smooth finish on vascular lumen	Require good surface finish to obtain smooth perfusion channel walls limiting blood damage and cell adhesion.
Aseptic handling	Minimal number of parts	Minimize sites where contamination may be introduced through leaks or air entry points.
	Minimal tools required for assembly	Easily assembled by a single person using sterile forceps.
	Minimal contact required for assembly	Minimize the area contacted with gloves or tools during assembly, particularly on interior surfaces (i.e., surfaces in contact with gel or fluid).
Functionality	Filling mechanism suitable for cell clusters	Filling ports must be large enough to accommodate cell clusters up to 500 μm in diameter suspended in alginate without damage or dispersion of the clusters.
	Maximize use of standard fittings and sizes	Device should accommodate widely available standard fittings to facilitate adaptation of connector sizes.
Size	Accommodate required number of islet equivalents (IEQ)	Intraportal infusion typically requires >10,000 IEQ/kg patient body weight (Hering et al., 2016), amounting to 600,000–800,000 IEQ in an average adult human or 3,000–5,000 IEQ in an average male rat.
Fluid mechanics	Withstand desired flow rate	Arteries > 100 μm diameter; 20–50 cm/s velocity; Cyclic blood pressure up to 300 mmHg; Cyclic wall shear stress up to 200 dyne/cm^2^ (Nichols et al., 2011).

The device size also provides space to house complex vascular networks, including branched geometries, while accommodating mm-scale gel thickness. For this initial study, a straight, stainless steel metal rod (1.6–2 mm) was selected as the vascular template to mimic the size of a medium to small artery, allowing for 5.4–5.6 mm of cell-laden gel between the perfusion channel and the device wall (Nichols et al., 2011). This simple geometry facilitates future investigation into the relationship between cell viability and proximity to the perfusion channel at various environmental oxygen tensions.

### *In vitro* Blood Compatibility

**FIGURE 2:**
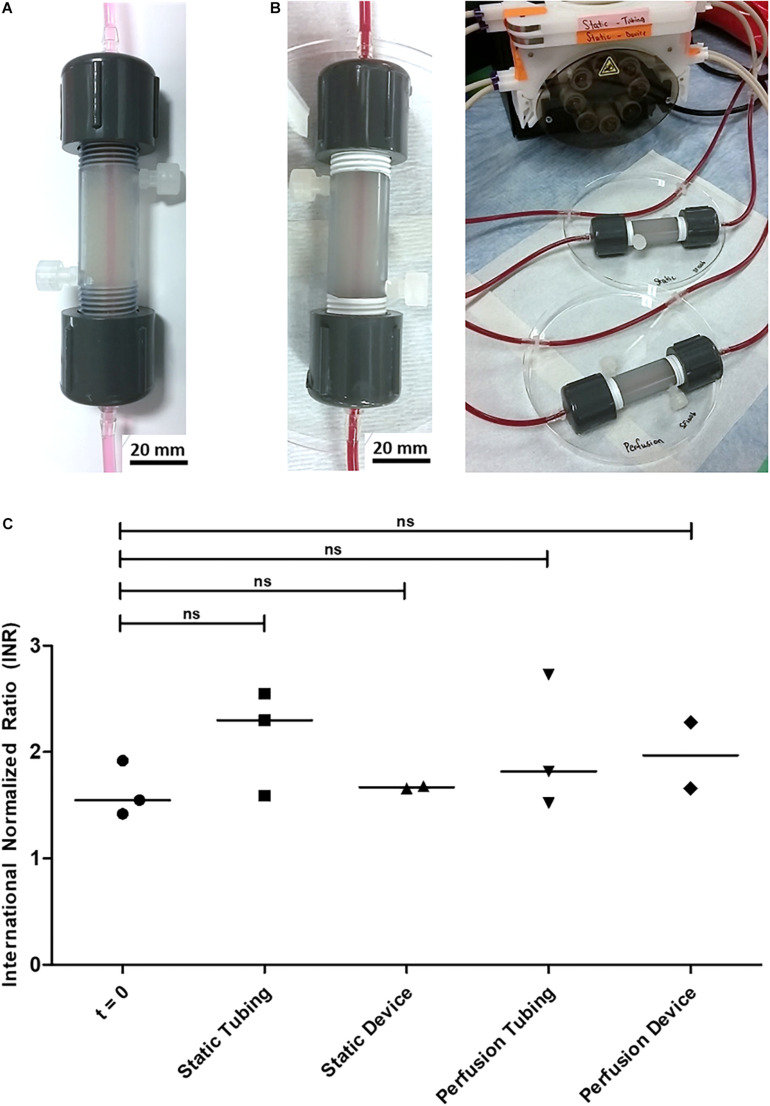
*In vitro* perfusion through Device 1 cast with 2% alginate and featuring a 2-mm channel for proof of concept. **(A)** Multi-day perfusion of cell culture medium (no cells encapsulated) at room temperature and at 37°C. **(B)** 2-h perfusion of citrated rat blood (no cells encapsulated) at room temperature. **(C)** International Normalized Ratio (INR) of citrated rat blood under static and perfusion conditions. Original blood samples are grouped under *t* = 0. The sample size is *N* = 3 for the *t* = 0, Static Tubing, and Perfusion Tubing conditions. *N* = 2 for the Static Device condition, which returned no value from the prothrombin time test performed during the second trial. Similarly, *N* = 2 for the Perfusion Device condition, which returned no value from the prothrombin time test performed during the third trial. From the Kruskal-Wallis and Wilcoxon-Mann-Whitney statistical tests, no significant difference was observed between the medians of the five sample groups. *P*-values of each condition compared with *t* = 0 are: 0.2000 (Static Tubing), 0.8000 (Static Device), 0.7000 (Perfusion Tubing), and 0.4000 (Perfusion Device).

To assess perfusability, Device 1 cast with 2% alginate hydrogel was connected to a tubing loop and perfused with fluid using a peristaltic pump. A low volumetric flow rate of 3 mL/min was selected to mimic laminar flow through a medium to small artery (Nichols et al., 2011), producing a Reynold’s number, Re <50 and shear rate 64 s^–1^. DMEM (with 10% FBS), which has a slightly higher viscosity than water (Wang et al., 2012; Fröhlich et al., 2013), was successfully perfused through the device both on the benchtop at room temperature and in an aseptic incubator at 37°C for up to 7 days with no observed leaking, contamination, or occlusion of the perfusion channel (Figure 2A). Channel diameter was measured immediately after alginate gelation (*t* = 0) as well as after 48-h perfusion with DMEM in a 37°C incubator to monitor changes caused by hydrogel swelling (Supplementary Figure 1). The mean ratio, t0/t48, was found to be 1.13 (SD 0.13), indicating a slight decrease in channel size after perfusion.

The interaction of the device with blood was tested using citrated rat blood collected from male Lewis rats (Figure 2B). Perfusion and static conditions were run for 2 h at room temperature with no visible diffusion of erythrocytes into the alginate (Figure 2B). To determine the effect of the encapsulation device and alginate gel on flow patency, plasma samples were analyzed for prothrombin time and international normalized ratio (INR) as presented in Figure 2C. No significant difference was observed between the INR values of the blood at time zero and after 2 h at any of the conditions. A significant decrease in clotting time would have been an early indication of a lack of hemocompatibility.

### *Ex vivo* Rat Studies

To assess device perfusability in a more physiologically relevant setup, animal studies were performed using male Lewis rats. These experiments were performed *ex vivo*, as internal device volumes were scaled for human therapeutic doses and downscaling would lead to changes in flow and mass transfer that do not reflect anticipated applications. Device 2 was cast with cell-free 2% alginate featuring a 1.6-mm perfusion channel. The flow loop was similar to the *in vitro* loop, substituting the peristaltic pump and perfusate reservoir with a live rat, allowing for observation of both device hemocompatibility and surgical considerations. These tests were designed to observe the magnitude and consistency of blood flow rate, as well as any possible coagulation or failure of the device. The desired observation time was at least 1 h to observe any severe adverse effects of the device on the animals and was extended to 2 h when possible. For ethical considerations, the observation time for each trial varied with the recommendation of the veterinary surgeon, depending on the time and difficulty of the surgical cannulation.

**FIGURE 3:**
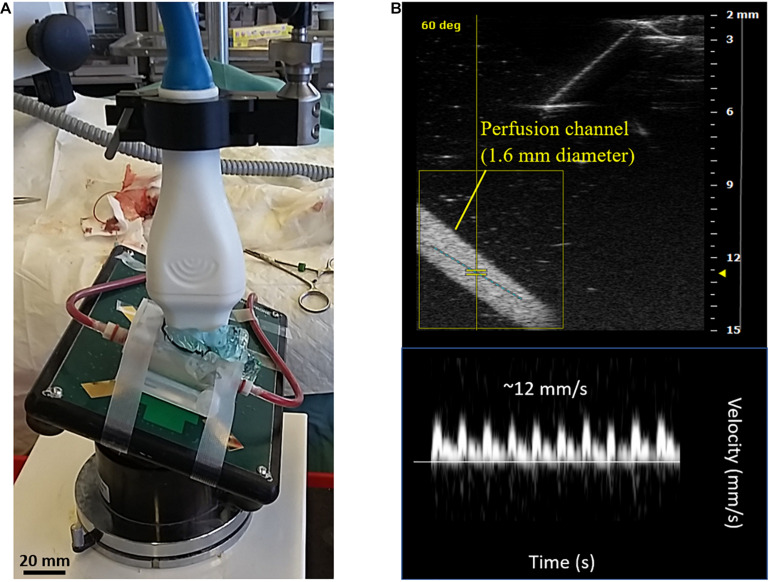
*Ex vivo* rat studies using perfusion devices cast with cell-free alginate. A total of 7 animals were tested with the perfusion device and one animal was tested with a tubing-only flow loop. **(A)** Perfusion setup with Device 2 and flow loop connected to the femoral artery of a male Lewis rat. The device aperture was opened to apply ultrasound gel directly onto the alginate. A transducer was inserted to record Doppler ultrasound images of the blood flow through the device. **(B)** Doppler ultrasound image of the 1.6-mm diameter perfusion channel (top) and velocity profile (bottom) recorded during the first *ex vivo* trial. The velocity profile corresponds to the peak values observed during the trial (approximately 12 mm/s or 1.5 mL/min).

No visible coagulation or channel occlusion was detected in the perfusion loops. As a supplement to the previous *in vitro* blood perfusions performed, one animal was connected to a tubing-only loop (no perfusion device). It exhibited no adverse reaction and performed similarly to animals connected to a perfusion device. A decrease in flow rate over time was observed due to failure of the animals rather than failure of the device. Failure of the animal was characterized by decreased heart and respiration rates, signs of pallor, and/or hypothermia, and was attributed to the stress on the animals, who underwent surgery for 1–2 h before connecting the perfusion loop. During the first *ex vivo* trial, Doppler ultrasound imaging was successfully performed through the Device 2 custom aperture up to a depth of 12 mm (Figure 3A). Blood flow velocities were monitored over a period of 105 min until failure of the animal, with peak values falling near 12 mm/s (1.5 mL/min), as shown in Figure 3B.

### Assessment of MIN6 Metabolic Activity and Apoptosis

**FIGURE 4:**
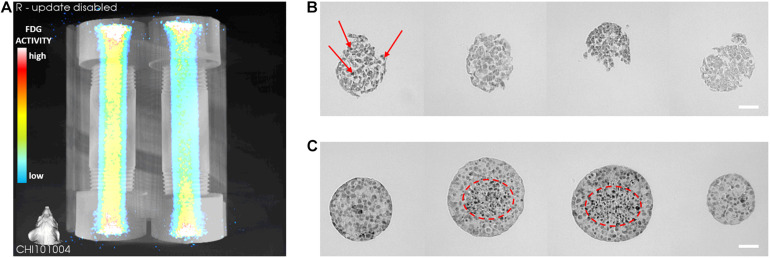
Preliminary metabolic activity and apoptosis of macroencapsulated MIN6 islet-like clusters (ILCs). **(A)** Three-dimensional PET-CT reconstruction of fludeoxyglucose (FDG) activity in alginate-encapsulated MIN6 ILCs (left) and a cell-free alginate control (right). FDG incubation and imaging were performed after 3 days of perfusion culture. Higher FDG activity appears in yellow and lower activity appears in blue. In the trial shown, FDG activity is 24% higher in the cell-laden device than in the cell-free control. Four pairs of devices were prepared from independent cell stocks for *N* = 4. **(B,C)** Histology: Cleaved caspase-3 staining of alginate-encapsulated MIN6 ILCs cultured under static **(B)** and perfused **(C)** conditions. Histological staining was performed after 7 days of culture. Three histological sections were stained with cleaved caspase-3 and imaged per device. Positive cleaved caspase-3 staining appears as black dots (examples indicated with red arrows or dashed circles). Three static and three perfused devices were prepared from independent cell stocks for *N* = 3 per condition. Scale bar = 100 μm.

A PET-CT imaging method was developed to visualize metabolically active MIN6 ILCs encapsulated in Device 1. A density of 1,000 beta cells per ILC was selected to mimic the beta cell content in native human islets (Pisania et al., 2010). Figure 4A shows a three-dimensional reconstruction of fludeoxyglucose (FDG) activity from one of four independent experimental trials. Areas of lower FDG activity appear in blue and areas of higher activity appear in yellow. The left-hand device corresponds to ILCs in alginate cultured under perfusion conditions for 3 days, while the right-hand device corresponds to a cell-free alginate control. The cell-laden device exhibits a color gradient (yellow to green to blue) from the perfusion channel to the device wall, indicating higher MIN6 metabolic activity detected near the perfusion channel. Some background activity (green/yellow regions) is observed in the cell-free control due to residual unbound FDG present in the gel after incubation and flushing. Across the four trials, FDG activity was 24–32% higher in the device containing ILCs than in the cell-free control (see Supplementary Figure 2). Areas of higher activity were observed in all devices near the inlet and outlet (yellow/red/white regions), which was associated with buildup of unbound FDG in the device caps.

To investigate the effect of fluid convection on cell apoptosis, MIN6 ILCs were encapsulated in Device 1 and cultured under perfusion and static conditions for 7 days. With static culture (Figure 4B), virtually all imaged cell clusters (*n* > 100 ILC sections) exhibited an abnormal morphology, with clusters breaking apart into irregular shapes. Apoptotic cells were detected at both the center and edges of the ILCs (examples of positive staining are indicated with red arrows in Figure 4B). Size distributions and mean staining intensities were not calculated for static samples due to pervasive ILC breakage. Under perfusion conditions, similarly irregular clusters were observed near the gel periphery (*n* > 50 ILC sections; difficult to accurately count due to ILC breakage). However, near the perfusion channel, a greater proportion of ILCs remained intact with a rounded morphology (*n* = 101 ILC sections; Figure 4C). Over 70% of intact ILCs increased in size from 100–300 μm upon harvesting to over 400 μm at day 7 post-encapsulation, which was not observed in the static condition. Further, over 25% of the intact ILCs were larger than 500 μm (Supplementary Figure 3A). Approximating these ILCs as spheres initially 150 μm in diameter, this corresponds to a 37-fold increase in ILC volume. The resulting growth rate of these ILCs near the perfusion channel is 0.52 day^–1^, which is higher than what we have previously observed in MIN6 adherent cultures (0.39 ± 0.03 day^–1^) and MIN6 cells encapsulated in 2% alginate microbeads (0.19 ± 0.02 day^–1^) (Hoesli et al., 2011). While calculating growth rate based on aggregate size may lead to an overestimation, as it excludes analysis of any single cells seeded, this observation suggests that aggregate growth was not impeded in this system. Analysis of cleaved caspase-3 staining intensity indicates that positive staining is significantly higher at ILC diameters >350 μm, as shown in Supplementary Figure 3B. This agrees with the visual observation of apoptotic cells present at the core of the clusters (circled in red in Figure 4C).

## Discussion

This work demonstrates hemocompatibility and cell survival in a macroencapsulation device developed for *in vitro* (Device 1) and *ex vivo* (Device 2) perfusion culture. The proposed system offers a way to accommodate thick cell-hydrogel constructs while establishing convective mass transport, which is currently lacking in the 3D cell culture field. The device is easily sterilized and assembled, leak-proof, and suitable for multi-day culture while maintaining aseptic conditions and flow patency. The device is resistant to relatively high flow rates—no leaks were observed up to flow rates of 100 mL/min. This parameter can therefore be adjusted to generate a range of shear rates as desired to mimic different blood vessels. It will also be important to characterize hydrogel swelling with different perfusates and flow conditions, particularly when designing for *in vivo* animal trials. With DMEM perfusion tests, the change in channel diameter associated with hydrogel swelling remained consistent, which allows for designing around this value in future work. In these studies, perfusion was flow-driven; however, it is possible to monitor the pressure in this system by introducing a pressure transducer at various points along the perfusion loop, such as the device inlet and outlet to assess the pressure drop. Pressure may also be adjusted by incorporating a resistance valve. While alginate-immobilized culture of ILCs has previously been achieved using hollow fiber bioreactors (HFBRs; Hoesli et al., 2009) and custom flow systems, to our knowledge this is the first time this is achieved without the presence of potentially thrombogenic polymeric membranes at the alginate/flow interface. Further, this device can be customized with different vascular geometries, while HFBRs are limited to straight perfusion channels.

Alginate is viewed as an anti-thrombogenic material due to its hydrophilicity, which lowers cell adhesion and protein adsorption (de Vos et al., 2006). While alginate has been used to engineer small-diameter vascular prostheses (Akentjew et al., 2019), its hemocompatibility has not been thoroughly studied in controlled *in vitro* settings. Typically, alginate-based vascular devices contain reinforcing polymers, which may not be needed at the higher alginate densities and volumes applied in closed devices as shown here (Hoesli et al., 2009). Using Device 1, prothrombin time and INR measurements indicated no significant change in clotting time of citrated rat blood running through the 2% alginate-cast device after 2 h (Figures 2B,C). Thus, no immediate concerns were raised with regard to the hemocompatibility of the alginate or the devices themselves in an *in vitro* setup. An *ex vivo* perfusion setup with Device 2 further demonstrated perfusability and short-term hemocompatibility of the device over a period >90 min as monitored using Doppler ultrasound imaging (Figures 3A,B). It is important to note that surgery was often challenging, causing extra stress on the animals and variability of perfusion times. This highlights a significant limitation of reliance on small animal models to predict *in vivo* performance in humans, particularly with intravascular device strategies. While surgery is expected to become easier in larger animal models, studies would become far more expensive. This reinforces the need for reliable *in vitro* platforms that can simulate *in vivo* conditions.

These preliminary results suggest that alginate hydrogel may be further studied as a possible hemocompatible bulk material for macroencapsulation devices. While we have observed long-term stability of internally gelled alginate microbeads over 2 weeks (Hoesli et al., 2012) to 5 months (unpublished) *in vivo*, next steps will assess alginate stability and degradation over longer time frames in the context of perfused vascular prostheses. Immune response may also be investigated within this system by perfusing various immune components such as antibodies, other proteins, and immune cells to monitor adhesion, adsorption, activation, and penetration into the hydrogel. Alginate porosity and permeability can be tuned by changing the alginate concentration and characterized through microscopy and chromatography techniques. Swelling of alginate in contact with blood will also be investigated, as the swelling ratio is expected to differ compared with DMEM perfusion conditions.

With respect to cell studies, the scale of the encapsulation device is an important design consideration. While rodent models are a popular choice to collect islet data, they pose several limitations. The size and resolution of rodent-scale devices are not suited to complex arrangements of cell clusters and artificial vasculature, nor to the optimization studies that would be required to predict performance in humans. As mentioned above, financial and ethical concerns would limit the use of rodent models and larger animal models, such as dogs or pigs, for extensive studies investigating oxygen effects. According to Papas et al. (2016), human-scale devices require a surface area on the order of 1,000 cm^2^ for adequate oxygenation via passive diffusion. Rather than using simplified animal models, it is therefore critical to design studies using human-scale devices to effectively investigate relevant oxygen profiles and optimize convective transport strategies.

The 3D reconstruction in Figure 4A and additional data in Supplementary Figure 2 shows that it is possible to identify metabolic activity of MIN6 ILCs encapsulated within a thick hydrogel using a PET-CT imaging technique. To our knowledge, this is the first report of human-scale pancreatic tissue construct imaging *in vitro*. Cell-laden devices consistently exhibited greater FDG activity than the cell-free controls, indicating the presence of metabolically active cells detectable via their uptake of radiolabeled FDG. The results also indicate that metabolic activity was detected at higher levels near the perfusion channel than near the device wall, as might be expected in the presence of an oxygen gradient (Fernandez et al., 2014). A limitation of this PET-CT method is a tendency for the unbound tracer molecule to become trapped in the hydrogel even in the absence of cells. Further optimization to increase the efficiency of the FDG incubation and flushing methodology could reduce the background signal in the hydrogel and allow for clearer detection of cell metabolic activity. Future studies may facilitate this optimization by incorporating multiple perfusion channels embedded in each device. This PET-CT technique could be valuable to obtain an overall picture of ILC metabolic activity after culturing under different oxygen conditions to complement histological staining. Additional techniques such as computational oxygen modeling could also be applied to correlate the FDG maps to the oxygen profiles within the devices.

Even with relatively low MIN6 seeding (1,000 ILCs/mL gel) and high environmental oxygen levels (21% O_2_ incubator), there was a distinct improvement in cell performance under perfusion conditions. This improvement is expected to be more drastic when assessing cell performance with higher seeding densities and under arterial oxygen conditions (9.5% O_2_), as would be most physiologically relevant for perfused vascular prostheses. As anticipated due to diffusion limitations, central necrosis was observed in larger ILCs (>350 μm diameter). This phenomenon has been predicted using theoretical models showing that oxygen transport by passive diffusion is more efficient in smaller clusters, in part due to a higher surface area-to-volume ratio (Dulong and Legallais, 2007; Fernandez et al., 2014; Papas et al., 2016). As the packing density of cell clusters increases within an encapsulation device, we can infer that oxygen transport by passive diffusion alone is insufficient to meet the demand. While oxygen content was not directly measured in the device for the purpose of this manuscript, it will be key for future in-depth investigation into oxygen effects on encapsulated beta cell performance.

Two prominent macroencapsulation strategies studied in recent clinical trials assessing safety and efficacy are the PEC-Encap^TM^ device developed by ViaCyte and the βAir device developed by Beta-O_2_ Technologies Ltd. (Agulnick et al., 2015; Carlsson et al., 2018). The PEC-Encap^TM^ consists of an immunoprotective material housing pancreatic progenitor cells and is intended for subcutaneous implantation (Agulnick et al., 2015). It is expected that multiple devices would be required to effectively treat a single adult human patient as this strategy relies on *in vivo* vascularization around the device to provide an interface for gas, nutrient, and insulin exchange. This process may take weeks to occur, leaving the implanted cells vulnerable to hypoxic effects immediately post-implantation. Another device by ViaCyte called the PEC-Direct^TM^ features materials that permit direct vascularization into the device to improve mass transfer (Cooper-Jones and Ford, 2016). However, this makes the therapeutic cells vulnerable to immune attack and once again necessitates immunosuppressive drugs. Beta-O2 takes an active oxygenation approach, proposing a device comprising both an islet macroencapsulation chamber and a gas chamber into which oxygen is supplied from an external source (Carlsson et al., 2018). While this is a promising attempt to improve cell viability by increasing the partial pressure of oxygen (pO_2_) in their device, negligible levels of C-peptide were detected in the circulation of device recipients in a recent Phase I clinical trial (Carlsson et al., 2018).

These challenges demonstrate the need for more rigorous testing and characterization of macroencapsulation devices in an *in vitro* setting. Establishing a perfusable macroencapsulation device as described in this manuscript allows for an in-depth examination of oxygen effects on human-scale doses of pancreatic beta cells. Future work entails culturing MIN6 cells at different oxygen levels to assess mass transport in normoxic, hypoxic, and physoxic environments. The vascular template will also be evolved to study the effect of more complex multivessel networks and optimize the oxygen distribution within the device. For example, this may be achieved by 3D printing sacrificial networks that are then embedded within the encapsulation matrix (Miller et al., 2012; Bégin-Drolet et al., 2017).

Keeping perfused vascular prostheses in mind, this system also permits an investigation into the hemocompatibility of alginate, including extended blood perfusion and coagulation studies, alginate degradation studies, and quantification of insulin secretion kinetics. Furthermore, alginate may be easily substituted to perform similar tests with other internally gelling or thermoresponsive polymers/composites, such as chitosan or polyethylene glycol (PEG). The matrix material may also be imbued with signal proteins such as vascular endothelial growth factor (VEGF) to promote vascular ingrowth.

Understanding the conditions influencing pancreatic beta cell survival and function in a three-dimensional environment is essential for developing effective therapeutic strategies for type 1 diabetes. The proposed *in vitro* system offers a robust and versatile platform that may be further developed in the long term to advance a variety of *in vivo* biomedical applications using convective mass transport.

## Data Availability Statement

The raw data supporting the conclusions of this article will be made available by the authors, without undue reservation.

## Ethics Statement

The animal study was reviewed and approved by the McGill University Animal Care Committee (UACC), McGill University and Affiliated Hospitals Research Institutes.

## Author Contributions

SF: conceptualization, methodology, validation, formal analysis, investigation, data curation, writing—original draft preparation, writing—review and editing, visualization, supervision, and project administration. LD: conceptualization, methodology, validation, investigation, writing—review and editing, and project administration. GG-R: software, formal analysis, investigation, data curation, writing—review and editing, and visualization. CH: conceptualization, methodology, and writing—review and editing. AB-D and JR: resources, writing—review and editing, supervision, and funding acquisition. SP: conceptualization, resources, writing—review and editing, supervision, and funding acquisition. RL and CH: conceptualization, methodology, resources, writing—review and editing, supervision, project administration, and funding acquisition. All authors contributed to the article and approved the submitted version.

## Conflict of Interest

The authors declare that the research was conducted in the absence of any commercial or financial relationships that could be construed as a potential conflict of interest.
