# Supplementary material for: An in vitro Perfused Macroencapsulation Device to Study Hemocompatibility and Survival of Islet-Like Cell Clusters
Source: Front Bioeng Biotechnol. 2021 May 28;9:674125. doi: 10.3389/fbioe.2021.674125 (PMC8193939; doi:10.3389/fbioe.2021.674125)
Supplement: Supplementary file 1 [file Data_Sheet_1.PDF]

Supplementary Material

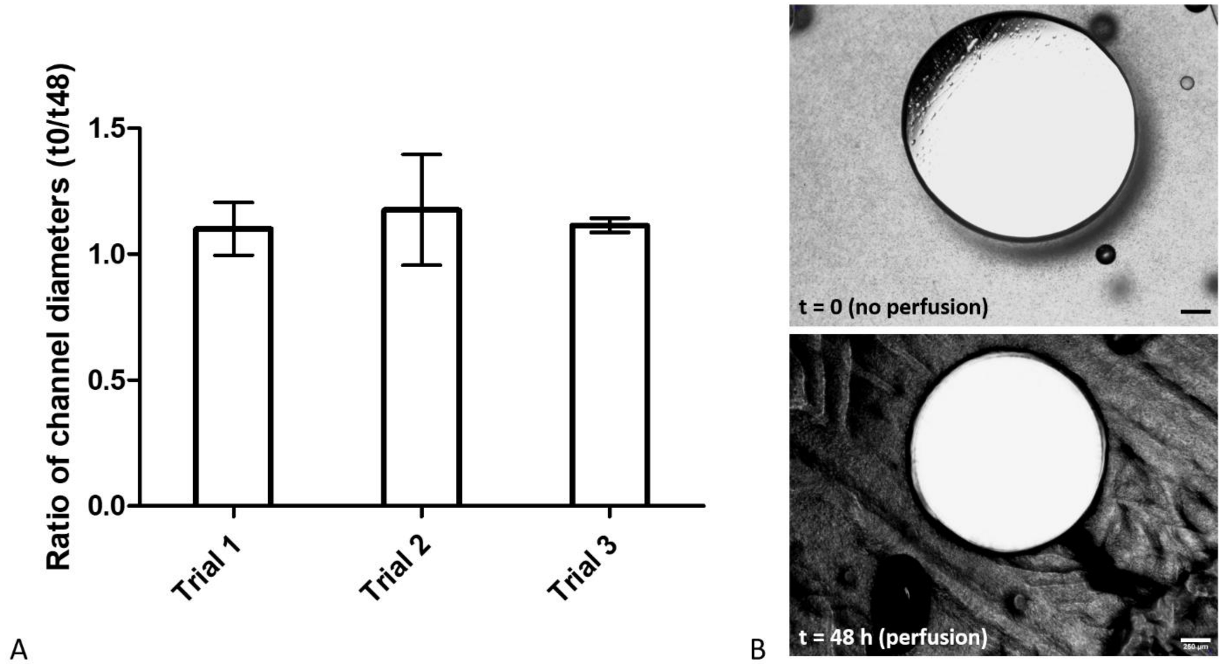

Supplementary Figure 1: Device channel diameter before and after *in vitro* perfusion with DMEM. Cell-free alginate was cast in Device 1 and the perfusion channel diameter was measured immediately after gelling (t = 0) and 48 h after perfusion with DMEM (t = 48 h) in a 37°C, 5% CO_2 incubator. **(A)** The ratio of the channel diameter after gelling to the diameter after 48-h perfusion (t0/t48). **(B)** Representative cross-sectional images of the perfusion channel at t = 0 and t = 48 h after perfusion. N = 3 independent trials (3 devices per condition). Three sections were analyzed per device: near the inlet, near the middle, and near the outlet of the device. From a one-way ANOVA (α = 0.05), no significant difference in channel diameter was observed across different positions within a single device. From a two-sample t-test assuming equal variances (α = 0.05), the overall mean diameter at t = 0 is significantly different from the overall mean diameter at t = 48 h (P = 0.0016). The mean ratio t0/t48 is 1.13 (SD 0.13). Error bars represent the standard deviation. Scale bar = 250 μm.

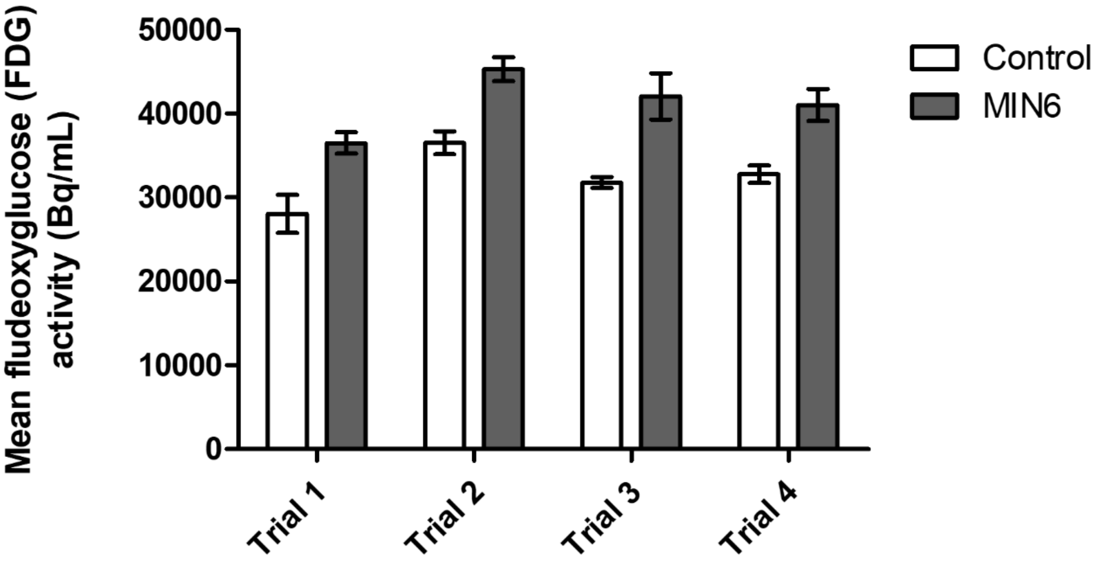

Supplementary Figure 2: Mean fludeoxyglucose (FDG) activity in perfusion devices. Cell-free (control) and cell-laden (MIN6) devices were imaged using PET-CT after 3-7 days of perfusion culture. Samples were incubated with FDG, a radiolabeled glucose analog that can be taken up by metabolically active cells. To analyze the FDG activity, sections were taken at 5-mm intervals orthogonal to the perfusion channel. Per sample, 9 sections (13 mm diameter, 0.4 mm thickness) were analyzed near the middle of the device to calculate the mean activity. Across four independent trials (N = 4), the FDG activity is 24-32% higher in the MIN6 devices compared with the controls. The mean relative increase is 28% (SD 4%). Error bars represent the standard deviation.

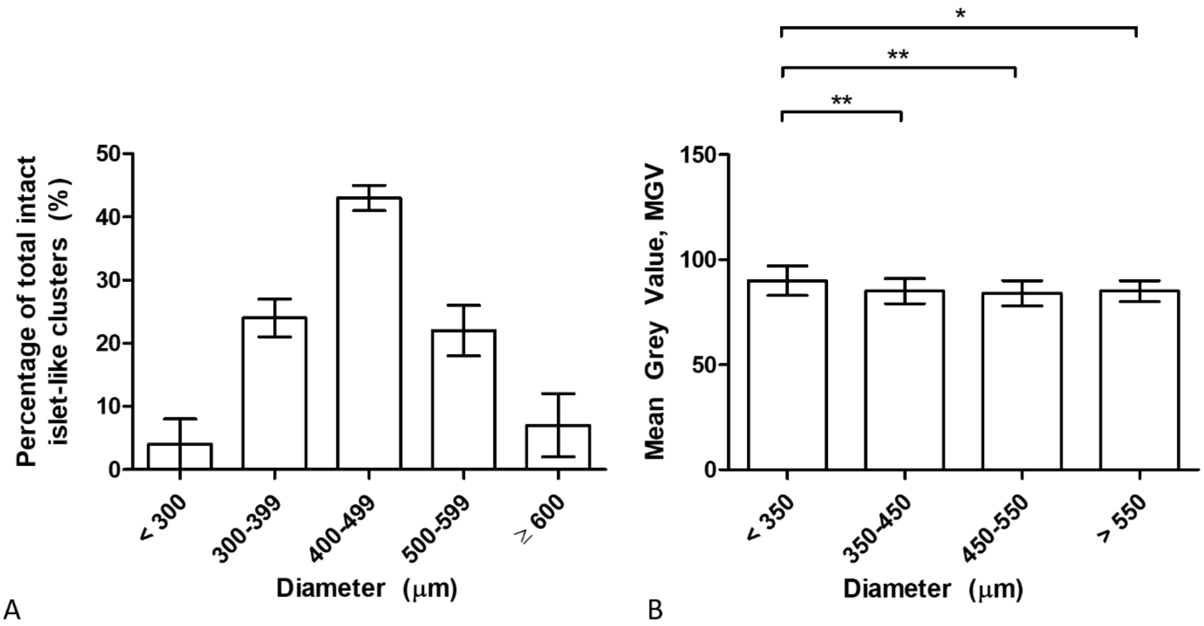

Supplementary Figure 3: Encapsulated MIN6 histology and staining analysis. MIN6 islet-like clusters (ILCs) were encapsulated and cultured in Device 1 for 7 days followed by histological staining. Under static culture conditions, virtually all ILCs were broken and consequently excluded from size distribution and staining intensity analysis. Under perfusion conditions, >50 ILC sections (located near the gel periphery) exhibited breakage, while 101 ILC sections (located near the perfusion channel) exhibited an intact morphology. The intact sections (n = 101) were further analyzed for size distribution and cleaved caspase-3 staining intensity. **(A)** Over 70% of intact ILCs increased in size from 100-300 μm upon harvesting to >400 μm in diameter at day 7 post-encapsulation, indicating cell proliferation. **(B)** Staining intensity was analyzed by calculating the mean grey value (MGV) for each intact section (n = 101). A lower MGV indicates higher positive staining and vice versa. Statistical analysis was performed using a one-way analysis of variance (ANOVA) and two-tailed t-test assuming equal variances (verified with an F-test). ILCs <350 μm in diameter exhibited significantly different MGVs compared with the 350-450 μm, 450-550 μm, and >550 μm ranges. *P = 0.0279; **P = 0.0051 and P = 0.0025. No significant difference was detected between any other pairs of conditions. N = 3 biological replicates. Error bars represent the standard deviation in both panels.
